# Supplementary material for: A Humanized Yeast Phenomic Model of Deoxycytidine Kinase to Predict Genetic Buffering of Nucleoside Analog Cytotoxicity
Source: Genes (Basel). 2019 Sep 30;10(10):770. doi: 10.3390/genes10100770 (PMC6826991; doi:10.3390/genes10100770)
Supplement: Supplementary file 1 [file genes-10-00770-s001.zip › Additional_Files/Additional_File7_GOTermHeatmaps.zip!/Additional_File7_GOTermHeatmaps/mitochondrial_fusion.pdf]

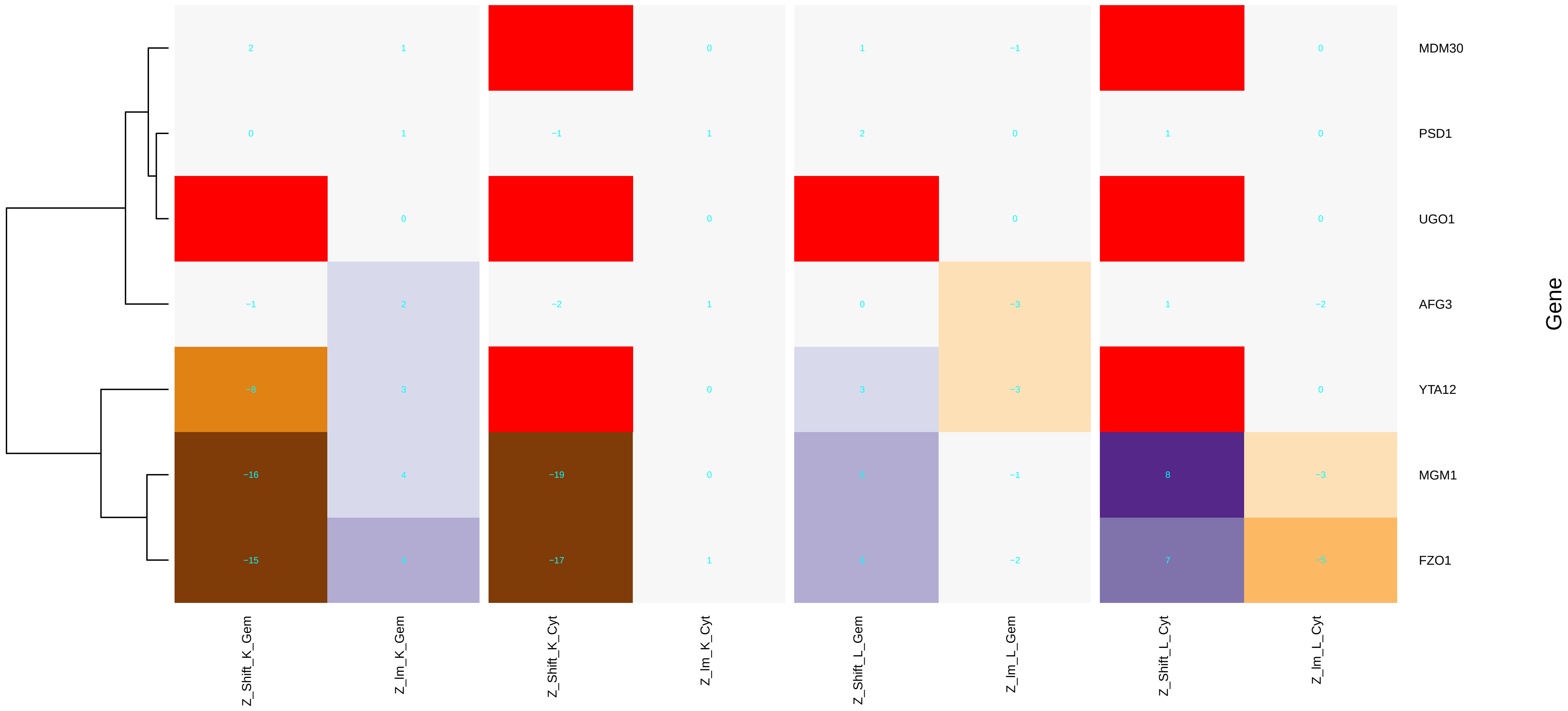

2
1
0
1
-1
0
0
1
-1
1
2
0
1
0
0
0
0
0
-1
2
-2
1
0
-3
1
-2
-8
3
0
3
-3
0
-16
4
-19
0
5
-1
8
-3
-15
4
-17
1
6
-2
7
-5
MDM30
PSD1
UGO1
AFG3
YTA12
MGM1
FZO1
Gene
Z_Shift_K_Gem
Z_Im_K_Gem
Z_Shift_K_Cyt
Z_Im_K_Cyt
Z_Shift_L_Gem
Z_Im_L_Gem
Z_Shift_L_Cyt
Z_Im_L_Cyt
